# Case report: Basivertebral nerve block during vertebral augmentation: an alternative approach to intraprocedural pain management

**DOI:** 10.3389/fradi.2023.1179023

**Published:** 2023-06-07

**Authors:** Giovanni C. Santoro, Siddhant Kulkarni, Diljot Dhillon, Kenny Lien

**Affiliations:** Department of Interventional Radiology, Mather Hospital, Northwell Health, Port Jefferson, NY, United States

**Keywords:** nerve block, kyphoplasty, vertebral augmentation, pain, anesthesia, vertebroplasty, vertebral augmentation

## Abstract

Osteoporotic compression fractures can be treated with vertebral augmentation. Since intraprocedural pain is common during vertebral body endplate manipulation, these procedures are often performed with conscious sedation or general anesthesia. Research has shown that vertebral endplates are innervated by the basivertebral nerve (BVN), which has been successfully targeted via radiofrequency ablation to treat chronic vertebrogenic lower back pain. With this physiology in mind, we evaluated if temporary BVN block would provide sufficient analgesia so that patients could forego sedation during percutaneous vertebral augmentation. Ten patients with single-level vertebral compression fractures were selected. Prior to balloon augmentation, temporary intraosseous BVN block was achieved using 2% lidocaine injection. All ten patients successfully completed their procedure without intraprocedural sedative or narcotic medications, and without significant deviation from baseline vital signs. Temporary BVN block can be used as intraprocedural anesthesia in select patients who may be poor candidates for general anesthesia or conscious sedation.

## Introduction

Vertebral compression fractures (VCF) of the thoracic and lumbar spine secondary to osteoporosis are often treated with percutaneous vertebral augmentation (PVA). Using fluoroscopic guidance, percutaneous access into the selected vertebral level is obtained via transpedicular or extrapedicular advancement of an introducer needle. Once within the vertebral body, a cavity is created, often by using curettage and balloon augmentation. Cement is then administered under fluoroscopic guidance through the introducer needle and into the cavity. During the procedure, the patient may experience significant pain due to vertebral body morphologic change and radiating pressure on the cortical bone of the superior and inferior endplates, particularly during balloon augmentation. Therefore, PVA is performed under conscious sedation in most cases, and under general anesthesia at certain institutions.

The overall risk for single-level treatment remains low to moderate (1, 2). However, there exists a subset of patients that would benefit clinically from augmentation, but are considered high risk candidates for anesthesia or sedation due to medical comorbidities. Contraindications to anesthesia, may result in certain VCFs being left untreated, which may have detrimental enduring effects. Long term studies have demonstrated that patients with VCF who do not receive PVA, have decreased mobility, increased morbidity and mortality, and an overall decreased quality of life (2–5). Therefore, it would be clinically valuable to reduce the procedural risk in this population, and offer vertebral augmentation to these patients.

The basivertebral nerve (BVN) innervates the superior and inferior endplates of the vertebral bodies (6–8). Recent histologic and clinical evidence suggests that axial load pain directed at the vertebral endplates is significantly reduced by ablation of this nerve (9). Permanent ablation of the BVN has proven successful in treating chronic vertebrogenic lower back pain via the Intracept® procedure (9, 10). Leveraging this knowledge, we postulated that temporary intraosseous BVN block with lidocaine could be used as an alternate intraprocedural analgesia during PVA. The goal of this study was to determine the feasibility of performing PVA without conscious sedation or general anesthesia, and instead by using only local anesthesia and temporary BVN block.

## Materials and methods

This study was performed at a single center community-based academic hospital interventional radiology practice, where vertebral augmentation is performed on a regular basis, and by a provider with extensive experience performing vertebral augmentation. Approval for human subjects research was obtained. Research was carried out in accordance with Institutional Research Board guidelines. A total of ten patients (five female, five male) between ages 50 and 90 years old were consented and enrolled in this study. All ten patients were of Caucasian ethnicity. Our inclusion criteria required that patients had a diagnosis of osteoporosis on dual energy x-ray absorptiometry (DEXA), had either an acute or subacute single level VCF between T10 and L3 as confirmed via magnetic resonance imaging (MRI) or nuclear medicine bone scan, and had initial pain score of greater than or equal to five upon initial consultation. Patients with vertebra plana compression fractures, burst fractures, and fracture with osseous retropulsion and associated severe spinal canal stenosis were excluded from the study. The pain score used was the standard clinical Visual Analog Scale (VAS) of 0–10, where zero indicates no pain and ten indicates the worst pain of the patient's life. Patients with psychiatric comorbidities, including anxiety, depression, and psychosis, were excluded from the study to mitigate the interaction of long-term sedative and anxiolytic medication use. Patients with a history of illicit drug abuse and/or alcohol abuse/dependence were excluded to minimize possible confounding effects due to altered nociception among these patients. Patient with Parkinson's Disease or other movement disorders were also excluded to minimize any potential confounding effects of dopaminergic medications.

All procedures were carried out by a single attending interventional radiologist. All patients received routine periprocedural clinical care, as well as additional intraprocedural monitoring for pain. During each procedure, local anesthesia was achieved using a subcutaneous injection of approximately 10 ml of 2% lidocaine and periosteal injection of 5 ml of 2% lidocaine solution, both via a 25 G needle. Thereafter, a dermatotomy was made and the affected vertebral body was accessed by advancing a 10-guage trocar introducer cannula (Kyphon T34A Express II Osteo Introducers) via a posterior transpedicular approach under continuous fluoroscopic guidance. The introducer needle was advanced incrementally under fluoroscopic guidance through the pedicle until the tip was just anterior to the posterior wall of the vertebral body, as confirmed on lateral imaging. The needle was then replaced with a 13-gauge curved cannula (16 cm straight, 3 cm curve, Medtronic Kyphon® Kurve Curved Bone Filler Device) and this was intermittently advanced under fluoroscopic guidance using alternating anteroposterior (AP) and lateral views, until the curved cannula was positioned at the expected anatomic location of the BVN.

**Figure 1:**
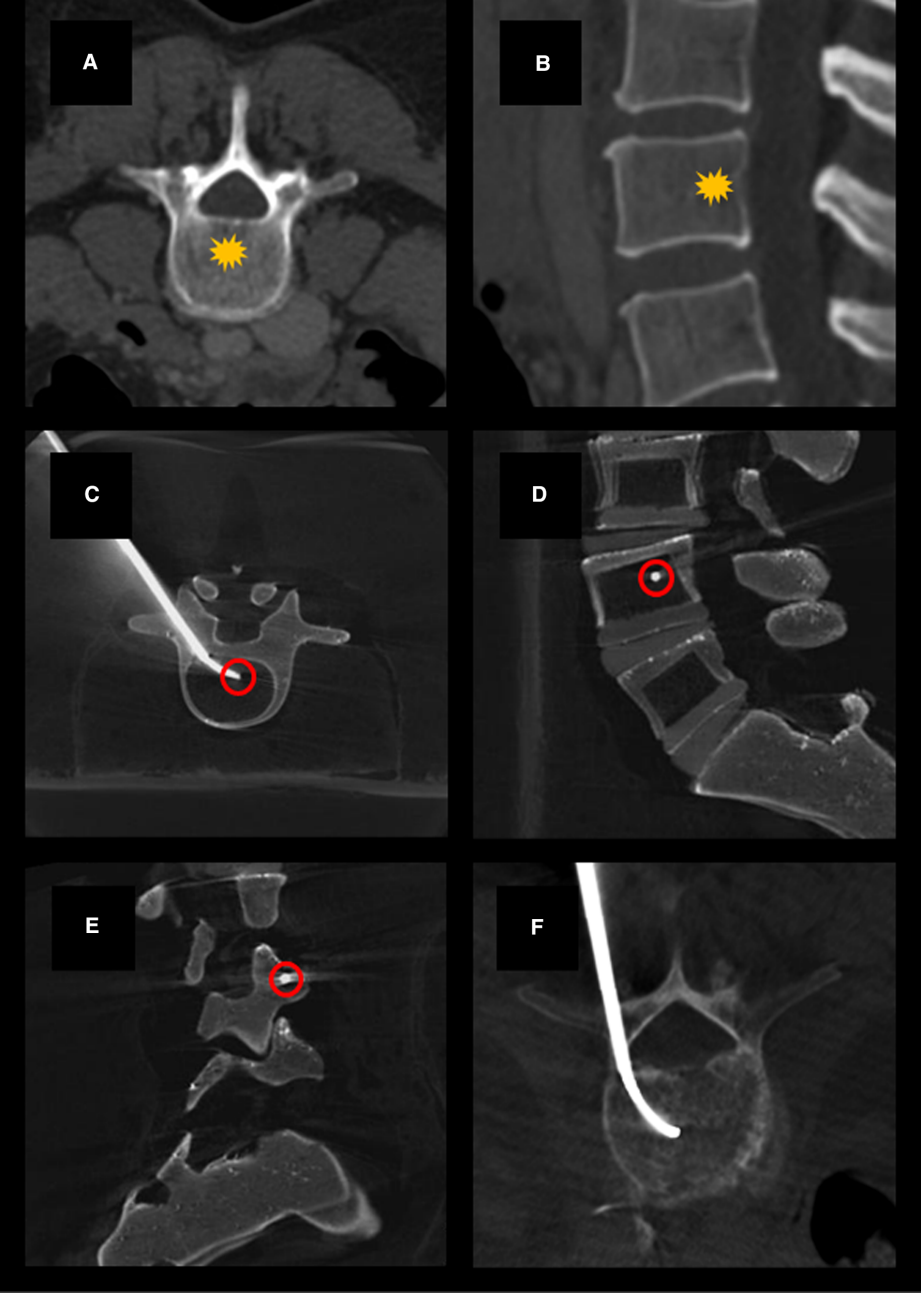
Example axial (**A**) and sagittal (**B**) CT images demonstrating normal vertebral bodies and the expected location of the basivertebral nerve (yellow star). Example cone-beam CT images acquired using a Medtronic Kyphon® training model showing transpedicular curved canula placement on axial (**C**), lateral (**D**), and oblique lateral (**E**) views. Canula tip placement (red circles) is ideally centrally located within the vertebral body on AP imaging and between 30% and 50% of the anterior-to-posterior length of the vertebral body from the posterior wall on lateral view. Example showing curved canula placement in a patient undergoing kyphoplasty with BVN block (**F**).

**Figure 2:**
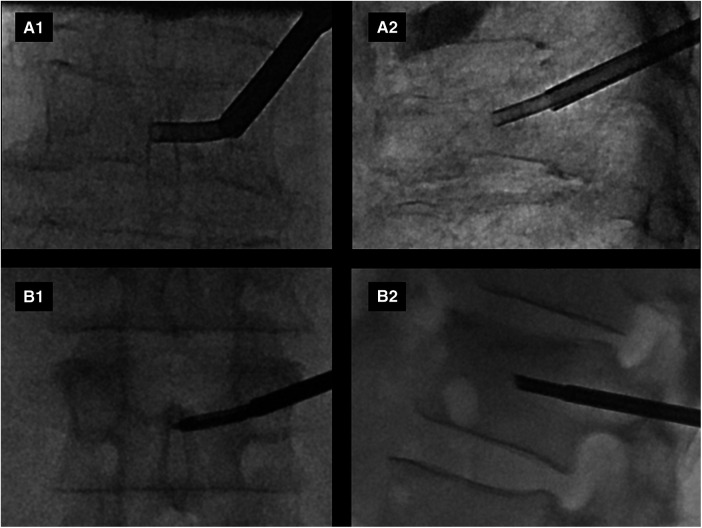
Example intraprocedural fluoroscopic images from two patients (**A**,**B**) enrolled in the study are shown. Anteroposterior (**A1**,**B1**) and lateral (**A2**,**B2**) views demonstrate curved canula positioning in the expected anatomic location of the basivertebral nerve plexus.

The positioning of the cannula was determined to be adequate once the distal tip was centrally located within the vertebral body on AP imaging and between 30% and 50% of the anterior-to-posterior length of the vertebral body from the posterior wall on lateral view (Figure 1). The position of the cannula tip at this anatomic location has been determined adequate for blockade of the basivertebral nerve (9, 10). Once positioning was confirmed fluoroscopically (Figure 2), the inner trocar was removed and an intraosseous injection of 5 ml of 2% lidocaine solution was administered via stylet. Contrast-lidocaine admixture for fluoroscopic localization was avoided, as this would preclude later visualization of the cement deposition. After this temporary BVN block was performed, vertebral augmentation was carried out using a routine unipedicular approach.

The curved cannula device was removed from the introducer, and a straight bone drill was advanced under fluoroscopy until the tip was located within the midline anterior vertebral body, as confirmed on alternating later and AP imaging. The drill was then removed, and a curetting device (Kyphon® Express Curette) was advanced and used to widen the drilled path in the medial, superior, and inferior dimensions. The curette was then removed and replaced with a balloon augmentation device (Kyphon® 15 mm Inflatable Bone Tamp KE152 balloon), which was expanded under fluoroscopic guidance. During the time period of balloon augmentation, all patients were verbally assessed for pain using the VAS, and vital signs (blood pressure and heart rate) were actively recorded. This time period was defined as the time beginning with balloon inflation and ending with balloon deflation and removal of balloon augmentation device.

During balloon augmentation, the polymethylmethacrylate (PMMA) bone cement (Kyphon® XPEDE Bone Cement and Mixer) was prepared on the back table. Following four minutes of cure time, the cement was visually inspected for the desired viscosity per the operator's preference. When the cement viscosity was deemed adequate, the balloon was deflated and removed from the introducer. The cement was then preloaded into two cartridges and attached to a delivery system with a curved bone filler (Kyphon® CDS CC02A, Kurve Bone Filler, CDS Size 2 BFDs CDS2A). Initial cement delivery was performed by advancing the curved filler into the introducer with fluoroscopic direction of the curved cannula to the contralateral side wall of the vertebral body across the midline. Incremental cement delivery was then performed under fluoroscopic control directing the cement into the known fracture planes. Once adequate cement deposition was provided to the contralateral portion, the curved cannula was retracted into the ipsilateral portion and further cement delivery was administered at the ipsilateral side of the vertebral body until adequate cement deposition into the fracture planes was achieved. If there was extravasation of cement visualized during the cement delivery, further administration was halted to ensure there was no migration of cement. Of note, in all these cases the volume of cement used was ≤4 ml (Table 1). This amount is less than has been previously reported (11). This decision was based on the performing radiologist's experience and personal preference. Post intervention cone beam computed tomography was performed post intervention to ensure adequate distribution of PMMA cement and for documentation purposes, per our departmental routine.

**Table 1:** Summary table includes relevant patient demographics.

Patient	Sex	Fx level	PS (pre)	PS (post)	PS (1 week)	SS (post)	SS (1 week)	BP/HR Δ	Balloon pain	Cement vol. (ml)
1	FM	L1	8	0	0	4	4	None	None	3.5
2	FM	T11	10	0	0	4	4	None	None	1.5
3	FM	T10	8	0	3	4	4	None	None	3.5
4	FM	T11	9	0	0	4	4	None	None	4
5	FM	L2	8	3	2	4	4	None	None	3
6	M	L3	5	0	0	4	4	None	None	3.5
7	M	L1	8	0	2	4	4	None	None	3
8	M	L1	6	0	0	4	4	None	None	3.5
9	M	L5	9	0	0	4	4	None	None	3.5
10	M	L2	5	0	2	4	4	None	None	3.5

Pain scores (PS) were obtained during preoperative consultation (pre), during the immediate postoperative period (post), and during the patient's one-week clinical follow-up (1 week). Satisfaction scores (SS) were also obtained postoperatively (post) and at one-week follow-up (1 week). Routine intraprocedural monitoring of vitals was carried out. There were no disturbances in patient blood pressure or heart rate as defined as >20% deviation from baseline (BP/HR Δ). Intraprocedural pain monitoring was routinely carried out, with special attention to intraprocedural balloon augmentation (Balloon Pain). No significant pain was reported by any patient during balloon inflation. Intravertebral cement injection was carefully carried out under fluoroscopic guidance to monitor for potential extravasation. The total volume of cement administered to each patient was recorded (Cement Vol.).

In addition to intermittent intraprocedural pain monitoring, patient pain was assessed using the VAS in the preoperative period, in the immediate postoperative period (following transfer of the patient from the fluoroscopy suite to the transport stretcher) and during routine 1 week follow up in clinic. Patients were also asked to rate their satisfaction using a subjective Likert scale of 1–4, at the same time intervals. Satisfaction ratings were defined as follows: 1 = completely dissatisfied, 2 = dissatisfied, 3 = satisfied, 4 = very satisfied. Upon discharge, patients were not prescribed opioid pain relievers by the interventional radiology medical staff. Patients were counseled to use over-the-counter analgesics, such as acetaminophen or nonsteroidal anti-inflammatory drugs, as needed.

## Results

All ten patients successfully underwent PVA using only local anesthesia and temporary BVN block with lidocaine as the primary intraoperative anesthesia. None of the patients enrolled in the study required additional analgesia or subsequent intraprocedural sedation. Routine monitoring of vitals did not show any significant alteration in blood pressure or heart rate, as defined as greater than 20% deviation from baseline.

Pain scores, satisfaction scores, and additional data are reported in Table 1. Nine of ten patients reported a complete reduction in pain immediately after their procedure. Only one patient reported 3/10 pain immediately post-procedure, which was overall reduced from the patient's baseline of 8/10. This patient subsequently demonstrated a further reduction in pain at one week, reporting 2/10 pain at that time. At one-week follow-up, one patient reported 3/10 pain and three patients reported 2/10 pain, while the remaining six patients reported no pain. Notably, all patients overall reported a decrease in pain score at one-week follow-up compared to their baseline at initial consultation. All patients reported a 4/4 (very satisfied) satisfaction score both immediately after their procedure and at one-week follow-up.

## Discussion

This prospective cases series suggests that blockade of the basivertebral nerve on a temporary basis is a safe and effective method of analgesia during vertebral augmentation procedures. This technique is translatable to other minimally invasive vertebral procedures, such as vertebroplasty without augmentation, as the steps for vertebral access and canula placement preceding cement injection are similar. The results of this study are consistent with previous research demonstrating the role of endplate related nociceptive sensation blockade via the basivertebral nerve.

Reviews comparing non-operative and operative management of vertebral compression fractures, demonstrate the efficacy of vertebral augmentation in reducing morbidity and mortality risk. Percutaneous vertebral augmentation is a minimally invasive procedure with relatively low rates of major complications. It is widely offered in many outpatient and inpatient settings. However, there is a subset of patients with vertebral compression fractures and comorbidities such as heart failure or severe emphysema, who do not undergo the procedure due to the increased risk associated with sedation or general anesthesia. We believe that by providing alternative intraoperative analgesia in these high-risk patients, we are better able to serve this population. Furthermore, beyond risk reduction in select patients, decreasing anesthesia requirements in during vertebral augmentation procedures can reduce cost to the healthcare system as well.

There are several inherent limitations to this study. Beyond appropriate canula placement at the expected location of the BVN, there was limited radiographic confirmation of lidocaine localization to BVN. In future studies, this could be addressed via injection of a small volume of contrast prior to lidocaine injection or alternatively a lidocaine/contrast admixture for localization. Additionally, although our study is limited due to sample size, the lack of control groups, limited, long-term follow-up, and potential confounding effects of periosteal lidocaine and PMMA administration, we were able to successfully eliminate the need for general anesthesia or conscious sedation in these select patients. This alone demonstrates that we can reduce the procedural risk associated with anesthesia in vertebral compression fracture patients, who are generally older and who often have multiple medical comorbidities.

To our knowledge, this is the first report where basivertebral nerve block has been used during vertebral augmentation. We hope that this positive experience will foster future research and reduce procedural risk, thereby increasing the quality of life among select patients with vertebral compression fractures.

## Data Availability

The original contributions presented in the study are included in the article, further inquiries can be directed to the corresponding author.

## References

[1] Beall DP Chambers MR Thomas S Amburgy J Webb JR Goodman BS Prospective and multicenter evaluation of outcomes for quality of life and activities of daily living for balloon kyphoplasty in the treatment of vertebral compression fractures: the EVOLVE trial. Neurosurgery. (2019) 84(1):169–78. 10.1093/neuros/nyy017 29547939 PMC6354561

[2] Marcia S Muto M Hirsch JA Chandra RV Carter N Crivelli P What is the role of vertebral augmentation for osteoporotic fractures? A review of the recent literature. Neuroradiology. (2018) 60(8):777–83. 10.1007/s00234-018-2042-0 29947942

[3] Buchbinder R Osborne RH Ebeling PR Wark JD Mitchell P Wriedt C A randomized trial of vertebroplasty for painful osteoporotic vertebral fractures. N Engl J Med. (2009) 361(6):557–68. 10.1056/NEJMoa0900429 19657121

[4] Piazzolla A Bizzoca D Solarino G Moretti L Moretti B. Vertebral fragility fractures: clinical and radiological results of augmentation and fixation-a systematic review of randomized controlled clinical trials. Aging Clin Exp Res. (2020) 32(7):1219–32. 10.1007/s40520-019-01289-1 31471888

[5] Sanli I van Kuijk SMJ de Bie RA van Rhijn LW Willems PC. Percutaneous cement augmentation in the treatment of osteoporotic vertebral fractures (OVFs) in the elderly: a systematic review. Eur Spine J. (2020) 29(7):1553–72. 10.1007/s00586-020-06391-x 32240375

[6] Bailey JF Liebenberg E Degmetich S Lotz JC. Innervation patterns of PGP 9.5-positive nerve fibers within the human lumbar vertebra. J Anat. (2011) 218(3):263–70. 10.1111/j.1469-7580.2010.01332.x 21223256 PMC3058212

[7] Fischgrund JS Rhyne A Franke J Sasso R Kitchel S Bae H Intraosseous basivertebral nerve ablation for the treatment of chronic low back pain: a prospective randomized double-blind sham-controlled multi-center study. Eur Spine J. (2018) 27(5):1146–56. 10.1007/s00586-018-5496-1 29423885

[8] Fischgrund JS Rhyne A Macadaeg K Moore G Kamrava E Yeung C Long-term outcomes following intraosseous basivertebral nerve ablation for the treatment of chronic low back pain: 5-year treatment arm results from a prospective randomized double-blind sham-controlled multi-center study. Eur Spine J. (2020) 29(8):1925–34. 10.1007/s00586-020-06448-x 32451777

[9] Becker S Hadjipavlou A Heggeness MH. Ablation of the basivertebral nerve for treatment of back pain: a clinical study. Spine J. (2017) 17(2):218–23. 10.1016/j.spinee.2016.08.032 27592808

[10] Khalil JG Smuck M Koreckij T Keel J Beall D A prospective, randomized, multicenter study of intraosseous basivertebral nerve ablation for the treatment of chronic low back pain. Spine J. (2019) 19(10):1620–32. 10.1016/j.spinee.2019.05.598 31229663

[11] Clark W Bird P Gonski P Diamond TH Smerdely P McNeil HP Safety and efficacy of vertebroplasty for acute painful osteoporotic fractures (VAPOUR): a multicentre, randomised, double-blind, placebo-controlled trial. Lancet (London, England). (2016) 388(10052):1408–16. 10.1016/S0140-6736(16)31341-1 27544377

